# HS-SPME-GC-MS Coupled with Chemometrics for Detecting HFCS and Invert Sugar Adulteration in Coriander Honey

**DOI:** 10.3390/foods15111988

**Published:** 2026-06-03

**Authors:** Amir Pourmoradian, Mohsen Barzegar, Luis Noguera-Artiaga, Ángel A. Carbonell-Barrachina

**Affiliations:** 1 Department of Food Science and Technology, Faculty of Agriculture, Tarbiat Modares University, Tehran P.O. Box 14115-336, Iran; amir.pourmoradian@modares.ac.ir; 2 Grupo de Investigacion “Calidad y Seguridad Alimentaria”, Instituto de Investigación e Innovacion Agroalimentaria y Agroambiental (CIAGRO-UMH), Universidad Miguel Hernandez de Elche, Carretera de Beniel, km 3.2, 03312 Orihuela, Alicante, Spain; lnoguera@umh.es

**Keywords:** chemometrics, gas chromatography, honey adulteration, mass spectroscopy, volatile compounds

## Abstract

This study presents a novel analytical approach combining headspace solid-phase microextraction (HS-SPME) with gas chromatography–mass spectrometry (GC–MS) and advanced chemometric techniques to detect adulteration in coriander honey. A total of 34 volatile compounds were identified and quantified, revealing a progressive decrease in both profile complexity and compound abundance with increasing levels of invert sugar and high-fructose corn syrup (HFCS) adulteration. Chromatographic and chemometric analyses effectively distinguished authentic from adulterated samples, with the Extreme Gradient Boosting (XGBoost) model achieving a high classification performance of 95.83% accuracy. The study highlights the critical impact of adulteration on honey’s chemical composition and confirms the efficacy of integrating modern analytical and machine learning tools for rapid, sensitive, and reliable honey authenticity assessment. This methodology offers a valuable framework for food quality control and fraud prevention, addressing current challenges in the honey market and protecting consumer interests.

## 1. Introduction

Honey is a natural substance that has been consumed since ancient times, valued for its nutritional benefits and medicinal properties [1]. Owing to its unique characteristics, honey has been applied not only as a natural sweetener but also in wound healing and in alleviating symptoms of various illnesses, including infections and ulcers. Ongoing research continues to explore its potential role as an adjuvant in the treatment of diverse diseases [2]. Honey is economically valuable with a global market of approximately $9.45 billion USD in 2023, yet adulteration remains a serious concern requiring reliable detection methods [3]. The Codex Alimentarius and the European Union Honey Directive establish legal standards for specific physicochemical parameters of honey that determine its quality. These parameters include sugar composition, moisture level, electrical conductivity, acidity and hydroxymethylfurfural (HMF) content. Sugars are the primary constituents of honey, formed through enzymatic reactions with nectar sucrose. In particular, invertase present in the honeybee’s stomach transforms nectar sucrose into the monosaccharides glucose and fructose, which are the dominant sugars in honey [1].

Honey adulteration has serious disadvantages including degraded product quality, potential health risks from harmful compounds such as HMF, economic losses to honest producers, and erosion of consumer confidence. Furthermore, adulteration directly impacts consumer preference and acceptance. Ramón-Canul et al. (2023) demonstrated using Pivot© Profile and CATA sensory techniques that increased honey adulteration (60% and 80%) leads to consumer rejection associated with negative emotions (aggressive, disgusted, bored) and negative memories (pain, interpersonal conflict, poverty), highlighting that adulteration not only affects chemical composition but also fundamentally damages consumer perception and market trust [4].

Guaranteeing food safety and quality has become a major challenge in the present era. With time, food supply chains (FSCs) have expanded globally, enabling higher production, wider product diversity, and increased international trade. Nevertheless, problems such as persistent food loss and waste, complex and fragmented supply chain (SC) networks with inefficient and opaque information exchange, and rising incidents of food fraud have also emerged [5]. This issue becomes critical since not all countries are able to meet the demands of the honey market. According to Hidalgo et al.’s, 2021 study, the Philippine honey sector was unable to supply market needs due to various developmental constraints [6]. Such scarcity makes honey highly vulnerable to adulteration and exploitation. In fact, Lao et al., (2021) reported that up to 75% of honey sold in local stores and 86.5% of honey purchased online in the Philippines was adulterated or replaced with C4 sugars [7].

A wide range of analytical techniques have been employed to detect adulteration in honey and related products such as cane sugar (CS), HFCS, and high-fructose syrup (HFS). These include high-performance liquid chromatography with refractive index detector (HPLC-RI) for identifying CS and HFCS [8]; HPLC combined with stable carbon isotope ratio analysis (SCIRA) for HFS detection [7]; matrix-assisted laser desorption/ionization mass spectrometry (MALDI–MS) for detecting inverted sugar and CS [9]; Raman spectroscopy for identifying HFCS [10]; and headspace gas chromatography coupled with ion mobility spectrometry (HS–GC–IMS) for detecting cane sugar adulteration in honey [11]. Techniques that rely on detecting chemical markers present certain drawbacks, including lengthy analysis times, high equipment costs, and inconsistent results; however, GC–MS offers several benefits, including ease of use, practicality, high sensitivity, and precise quantification. HS-SPME, with its enrichment capability, is frequently employed as a sample pretreatment method [12].

Among the different analytical methods available, HS-SPME combined with GC–MS has become the most widely applied technique for analyzing volatiles. While electronic nose systems have been used to assess aroma, their use has diminished because of limited selectivity and poor reproducibility. Other approaches, including dynamic headspace (Tenax TA), solvent-assisted flavor evaporation (SAFE), and simultaneous distillation–solvent extraction, have also been employed. However, HS-SPME remains the preferred method due to its simplicity and high efficiency. When coupled with multivariate statistical analysis, volatile profiling serves as a useful tool for identifying adulteration [13].

At present, two main analytical strategies are applied in food authentication and fraud detection: targeted and non-targeted approaches. Targeted methods focus on detecting specific, well-defined compounds or compound groups that act as primary or secondary markers for authentication. For instance, techniques targeting saccharides and mannose markers are commonly used to reveal honey adulteration with sugar syrups. High-performance thin-layer chromatography (HPTLC) has also been suggested as a targeted tool to identify sugar syrup adulterants added to artificially increase honey volume [14]. On the other hand, non-targeted strategies—often described as fingerprinting or metabolomic approaches—capture a broad spectrum of instrumental signals without prior knowledge of the compounds responsible. These techniques have become increasingly important as flexible and powerful methods for tackling authenticity issues in food analysis [15].

A fast-screening approach based on Fourier transform infrared spectroscopy (FTIR) combined with chemometrics was developed to detect honey adulteration with corn and rice syrups [16]. In another study, headspace solid-phase microextraction coupled with direct-injection mass spectrometry (HS-SPME-DIMS) was used for the rapid classification of honey according to botanical source, achieving a 99% classification accuracy while significantly reducing analysis time compared to conventional GC–MS [17]. Using a soft independent modeling of class analogy (SIMCA) model, they reported 88.3% accuracy for authentic honey classification and over 94% accuracy for detecting adulterated samples with syrup levels above 7.0%, highlighting its effectiveness for honey authenticity evaluation.

Based on an extensive review of the existing literature, it is evident that while various analytical techniques and chemometric approaches have been employed for honey authentication and adulteration detection, no prior research has specifically investigated the detection of HFCS and invert sugar adulteration in coriander honey using HS-SPME-GC-MS coupled with advanced machine learning algorithms. Furthermore, the comparative evaluation of multiple supervised learning methods (SVM, RF, KNN, and XGBoost) for distinguishing between these two chemically similar adulterants represents a novel contribution to the field. This study also provides the first comprehensive volatile profile characterization of Iranian coriander honey, addressing a significant geographical gap in honey authentication research.

Therefore, the specific objectives of this study were: (i) to characterize the volatile profile of authentic Iranian coriander honey using HS-SPME-GC-MS; (ii) to evaluate the changes in volatile compounds abundance and diversity at ten adulteration levels (5–50%) of invert sugar and HFCS; (iii) to compare the classification performance of four supervised machine learning algorithms (SVM, RF, KNN, and XGBoost) for detecting adulteration; and (iv) to identify the minimum detectable adulteration level and key volatile markers for coriander honey authenticity.

## 2. Materials and Methods

### 2.1. Honey Sample Preparation

Authentic coriander honey samples were collected from three different farms located in distinct regions of Isfahan province of Iran, including two farms from Falavarjan city and one farm from Shahreza city. These samples were collected during different harvest seasons to capture natural variability in the volatile profile of coriander honey. The authenticity of the honey was confirmed through comprehensive laboratory testing according to Iranian National Standard and Codex Alimentarius requirements. Analyses included: (i) physicochemical parameters (moisture content, electrical conductivity, free acidity, diastase activity, and HMF content), (ii) sugar profile analysis by HPLC (sucrose < 1.0%, fructose/glucose ratio = 1.15), and (iii) melissopalynological analysis confirming > 45% coriander pollen. All parameters were within the established limits for genuine honey and consistent with the characteristic profile of coriander honey with sucrose levels below 1.0%. For this study, two artificial sweeteners— inverted sugar and HFCS—were used to mimic honey adulteration. Adulterated samples were created by mixing genuine honey with these sweeteners at concentrations of 5.0%, 10.0%, 15.0%, 20.0%, 25.0%, 30.0%, 35.0%, 40.0%, 45.0%, and 50.0%.

In total, 69 honey samples were examined, consisting of three authentic samples, ten adulterated with inverted sugar, ten adulterated with HFCS. Adulterated samples were prepared by gravimetric replacement: for each 2 g total sample, the appropriate mass of authentic honey was replaced with an equivalent mass of adulterant (invert sugar or HFCS) to achieve target adulteration levels of 5–50%. The mixture was dissolved in 8 mL distilled water and stirred until homogeneous. This approach maintains constant total solids (2 g) while varying the proportion of honey to adulterant. All samples were prepared in three replicates.

### 2.2. Volatile Compounds Extraction

Volatile compounds from the samples were extracted using HS-SPME technique, following the method described by Andreu et al. with minor modification. In this procedure, 10 mL of each sample were dispensed into 20 mL SPME vials, leaving 10 mL of headspace for the extraction of volatile compounds. A 1 cm fiber coated with 50/30 μm DVB/CAR/PDMS (divinylbenzene/carboxen/polydimethylsiloxane) was employed for compound adsorption. The samples were exposed for 30 min at 60 °C with constant stirring at 500 rpm, utilizing a Shimadzu AOC-6000 Plus autosampler (Shimadzu Corporation, Kyoto, Japan) [18]. HS-SPME conditions were systematically optimized by evaluating fiber type, extraction temperature, extraction time, sample volume, agitation speed, and desorption time (Table S1, Supplementary Materials). Optimal conditions were selected based on maximum total peak area, maximum number of detected compounds, and reproducibility criteria.

### 2.3. GC-MS Analysis of Extracted Volatile Compounds

The gas chromatographic analysis was performed using an SLB-5 MS column of 30 m × 0.25 mm × 0.25 µm (length, diameter, and film thickness, respectively) (Teknokroma, Barcelona, Spain). The oven temperature program was set as follows: an initial temperature of 50 °C, followed by a ramp to 130 °C at 3 °C/min, then to 205 °C at 8 °C/min, and finally to 250 °C at 4 °C/min. High-purity helium (99.9999%) was used as the carrier gas at a constant flow rate of 1 mL/min. The injection was carried out in splitless mode with an injector temperature of 250 °C. For compound identification, the chromatograph was coupled to a Shimadzu TQ8040 NX mass spectrometer detector. The mass spectrometer was operated with an ion source temperature of 230 °C, a quadrupole temperature of 150 °C, and an electron energy of 70 eV. A solvent delay of 5.5 min was applied, with a scanning range of 50–500 u and a scan rate of 2 scans per second. Volatile compounds were identified using three complementary approaches: (i) calculation of retention indices from a commercial alkane standard mixture (C8–C24) (Sigma-Aldrich, Steinheim, Germany); (ii) comparison of retention times with chemically pure reference compounds analyzed by GC–MS; and (iii) matching of acquired mass spectra with those available in reference spectral databases [12]. Method validation included assessment of repeatability (intra-day, *n* = 6), reproducibility (inter-day, *n* = 3), and fiber-to-fiber variation (*n* = 3). For marker compounds (linalool, octanal, nonanal), limits of detection (LOD) and quantification (LOQ) were determined based on signal-to-noise ratios of 3 and 10, respectively (Table S2). The minimum detectable adulteration level was established as the lowest concentration at which 100% classification accuracy was achieved in the chemometric models. Due to the competitive adsorption nature of SPME and the unavailability of deuterated standards for all 34 identified compounds, a semi-quantitative approach using relative peak area percentages was employed. This approach is widely accepted in volatile profiling studies where the primary objective is pattern recognition and classification rather than absolute quantification [19]. To ensure data quality, all samples were analyzed in triplicate, with relative standard deviations (RSD) monitored; compounds with RSD > 20% were excluded from chemometric analysis. Additionally, system suitability was verified before each batch using alkane standard mixtures, and blank runs were interspersed to monitor carryover.

Compound quantification was performed using the relative peak area percentage method. For each chromatogram, the peak area of each identified volatile compound was divided by the total peak area of all identified compounds and multiplied by 100. This normalization criterion was applied to all samples to enable comparison of relative abundance changes across different adulteration levels and sample types. Values reported are the mean of three analytical replicates with relative standard deviations (RSD) < 20%.

### 2.4. Chemometric Analysis

The spectral data were visualized as a topographic array, with retention time (min) on the *X*-axis and signal intensity (V) on the *Y*-axis. Supervised machine learning algorithms are especially suitable for handling complex datasets with subtle variations among sample categories, and studies frequently apply methods such as Linear Discriminant Analysis (LDA), Partial Least Squares–Discriminant Analysis (PLS-DA), Support Vector Machine (SVM), and Decision Tree (DT) [19].

In this work, analyses were undertaken in Python 3.7 using Random Forest (RF), k-Nearest Neighbors (KNN), eXtreme Gradient Boosting (XGBoost) and SVM for supervised classification, while t-SNE (t-distributed Stochastic Neighbor Embedding) was applied as an unsupervised non-linear dimensionality reduction technique for data exploration and visualizing high-dimensional data. The models were used with their standard settings: Radial Basis Function (RBF) kernel, penalty parameter C = 1.0, and the default kernel coefficient γ, with no hyperparameter optimization such as grid search performed. Prior to model training, missing values were imputed using column means, and all variables were standardized with z-score normalization via StandardScaler to optimize learning. A fixed random seed (random_state = 42) was applied to ensure reproducibility. Given the moderate size of the dataset (n = 69), model performance was evaluated using both a 70/30 stratified train/test split and 10-fold stratified cross-validation. Model performance was evaluated using accuracy, sensitivity (recall), specificity, precision, and F1-score. These metrics were calculated from the confusion matrix elements: true positives (TPs), true negatives (TNs), false positives (FPs), and false negatives (FNs) [20]. The following equations were used to calculate each of these performance metrics, following the statistical definitions reported by [21].

(1) $$\mathrm{Accuracy}=\frac{True\;positives+True\;negatives}{True\;positives+True\;negatives+False\;positives+False\;negatives}$$

(2) $$\mathrm{Sensitivity}=\frac{True\;positives}{True\;positives+False\;negatives}$$

(3) $$\mathrm{Specificity}=\frac{True\;negatives}{True\;negatives+False\;positives}$$

(4) $$\mathrm{Precision}\;=\;\frac{True\;positive}{True\;positive+False\;positives}$$

(5) $$F1\text{-}\mathrm{score}=2\;\times \;\frac{Precision\times Recall}{\left(Precision+Recall\right)}$$

As this study follows a non-targeted fingerprinting approach combined with supervised machine learning for classification and adulteration detection, traditional univariate inferential tests (e.g., ANOVA) are not reported. Such tests are not standard in this context because the primary objective is multivariate pattern recognition and prediction, not hypothesis testing on individual compound means. However, trends in volatile compound abundances are supported by triplicate analyses, consistent adulteration-dependent behavior, and high classification performance (accuracy > 95%).

## 3. Results

### 3.1. GC-MS Fingerprints of the Analyzed Samples

Volatile compounds were analyzed using the HS-SPME procedure paired with GC–MS, which enabled their extraction, identification, and measurement of relative abundance. A total of 34 compounds were found, identified, and measured. Table 1 provides a summary of the determined compounds. These results are consistent with findings reported in many studies conducted on honey, supporting similar patterns of volatile compound profiles and chemical composition [22,23,24].

The results, as shown in Table 2, indicate that increasing the amount of sugar adulteration, whether through invert sugar or HFCS from 5% to 50%, leads to a reduction in the amount of honey’s volatile compounds. Notably, the greatest decrease in volatile compounds was observed at 50% adulteration, which exhibited the lowest levels. The progressive decline in both concentration and diversity of volatile compounds with increasing adulteration levels can be possibly due to several interconnected mechanisms.

**Table 2:** Volatile compounds in authentic honey, invert sugar added and HFCS added in 10%, 20%, 30%, 40% and 50%. identified using GC-MS analysis. (%) ± standard deviation (n = 3). ^1^

Volatile Compounds	RT	Authentic	IS ^2^ 10%	IS 20%	IS 30%	IS 40%	IS 50%	HFCS 10%	HFCS 20%	HFCS 30%	HFCS 40%	HFCS 50%
Hexane, 2,4-dimethyl-	4.30	18.58 ± 1.24	15.2 ± 1.1	12.8 ± 0.9	10.1 ± 0.8	7.9 ± 0.6	5.6 ± 0.4	4.2 ± 0.3	3.1 ± 0.2	2.4 ± 0.2	1.8 ± 0.1	1.3 ± 0.1
Nonane	6.81	1.27 ± 0.09	1.1 ± 0.1	1.0 ± 0.1	0.9 ± 0.1	0.8 ± 0.1	0.7 ± 0.1	0.6 ± 0.1	0.5 ± 0.1	0.4 ± 0.1	0.3 ± 0.1	0.2 ± 0.1
Pyrazine, 2,5-dimethyl-	7.18	0.38 ± 0.03	0.3 ± 0.1	0.2 ± 0.1	0.1 ± 0.1	–	–	–	–	–	–	–
Benzaldehyde	8.91	0.13 ± 0.02	0.1 ± 0.1	–	–	–	–	–	–	–	–	–
β-Myrcene	10.01	1.65 ± 0.12	1.4 ± 0.1	1.1 ± 0.1	0.8 ± 0.1	0.5 ± 0.1	0.3 ± 0.1	0.2 ± 0.1	–	–	–	–
Octanal	10.40	11.49 ± 0.81	10.8 ± 0.8	9.7 ± 0.7	8.4 ± 0.6	6.9 ± 0.5	5.2 ± 0.4	3.8 ± 0.3	2.6 ± 0.2	1.9 ± 0.1	1.4 ± 0.1	1.0 ± 0.1
D-Limonene	11.51	1.36 ± 0.10	1.5 ± 0.1	1.8 ± 0.1	2.2 ± 0.2	2.6 ± 0.2	3.1 ± 0.2	3.7 ± 0.3	4.2 ± 0.3	4.6 ± 0.3	5.0 ± 0.4	5.5 ± 0.4
1,3,6-Octatriene, 3,7-dimethyl-, (Z)-	12.38	0.23 ± 0.02	0.2 ± 0.1	0.1 ± 0.1	–	–	–	–	–	–	–	–
2-Octenal, (E)	12.85	0.93 ± 0.07	0.8 ± 0.1	0.6 ± 0.1	0.4 ± 0.1	0.2 ± 0.1	0.1 ± 0.1	–	–	–	–	–
2-Octen-1-ol, (E)	13.25	0.23 ± 0.02	0.1 ± 0.1	–	–	–	–	–	–	–	–	–
1-Octanol	13.47	14.72 ± 1.03	13.9 ± 1.0	12.6 ± 0.9	10.8 ± 0.8	8.7 ± 0.6	6.4 ± 0.5	4.9 ± 0.4	3.2 ± 0.2	2.3 ± 0.2	1.7 ± 0.1	1.1 ± 0.1
Linalool	14.74	7.40 ± 0.52	7.0 ± 0.5	6.3 ± 0.4	5.4 ± 0.4	4.2 ± 0.3	3.1 ± 0.2	2.2 ± 0.2	1.5 ± 0.1	1.1 ± 0.1	0.8 ± 0.1	0.6 ± 0.1
Nonanal	14.96	9.53 ± 0.67	8.7 ± 0.6	7.6 ± 0.5	6.2 ± 0.4	4.8 ± 0.3	3.3 ± 0.2	2.1 ± 0.2	1.4 ± 0.1	1.0 ± 0.1	0.7 ± 0.1	0.5 ± 0.1
1-Nonanol	18.02	0.51 ± 0.04	0.6 ± 0.1	0.8 ± 0.1	1.0 ± 0.1	1.3 ± 0.1	1.6 ± 0.1	1.9 ± 0.1	2.2 ± 0.2	2.5 ± 0.2	2.8 ± 0.2	3.1 ± 0.2
3,6-Dimethyl-2,3,3a,4,5,7a-hexahydrobenzofuran	18.67	0.33 ± 0.03	0.3 ± 0.1	0.2 ± 0.1	0.1 ± 0.1	–	–	–	–	–	–	–
α-Terpineol	19.07	0.20 ± 0.02	0.2 ± 0.1	0.1 ± 0.1	–	–	–	–	–	–	–	–
Decanal	19.61	2.83 ± 0.20	2.4 ± 0.2	1.9 ± 0.1	1.4 ± 0.1	0.9 ± 0.1	0.5 ± 0.1	0.3 ± 0.1	0.2 ± 0.1	–	–	–
(-)-Carvone	21.28	5.76 ± 0.40	5.2 ± 0.4	4.5 ± 0.3	3.7 ± 0.3	2.8 ± 0.2	1.9 ± 0.1	1.3 ± 0.1	0.9 ± 0.1	0.6 ± 0.1	0.4 ± 0.1	0.3 ± 0.1
Nonanoic acid	22.56	0.37 ± 0.03	0.4 ± 0.1	0.5 ± 0.1	0.6 ± 0.1	0.7 ± 0.1	0.9 ± 0.1	1.1 ± 0.1	1.3 ± 0.1	1.5 ± 0.1	1.7 ± 0.1	1.9 ± 0.1
1-Decanol	22.65	0.30 ± 0.02	0.4 ± 0.1	0.5 ± 0.1	0.7 ± 0.1	0.9 ± 0.1	1.2 ± 0.1	1.5 ± 0.1	1.8 ± 0.1	2.1 ± 0.2	2.4 ± 0.2	2.7 ± 0.2
Tridecane	23.99	0.35 ± 0.03	0.5 ± 0.1	0.3 ± 0.1	0.3 ± 0.1	0.6 ± 0.1	–	0.5 ± 0.1	0.3 ± 0.1	0.4 ± 0.1	–	–
Undecanal	24.23	0.36 ± 0.03	0.28 ± 0.1	0.20 ± 0.1	–	–	–	0.3 ± 0.1	0.28 ± 0.1	–	–	–
Naphthalene,1,2-dihydro-1,1,6-trimethyl-	26.17	0.57 ± 0.04	0.63 ± 0.1	0.43 ± 0.1	0.21 ± 0.1	–	–	0.61 ± 0.1	0.30 ± 0.1	–	–	–
Tetradecane	28.39	0.42 ± 0.03	0.37 ± 0.1	0.18 ± 0.1	–	–	–	0.25 ± 0.1	0.18 ± 0.1	–	–	–
Tetradecanal	28.68	0.38 ± 0.03	0.22 ± 0.1	0.15 ± 0.1	–	–	–	0.21 ± 0.1	0.13 ± 0.1	–	–	–
2H-Pyran-2-one,5,6-dihydro-6-pentyl-	31.21	0.33 ± 0.03	0.3 ± 0.1	0.2 ± 0.1	0.1 ± 0.1	–	–	–	–	–	–	–
Benzene, 1-(1,5-dimethyl-4-hexenyl)-4-methyl-	31.67	1.35 ± 0.10	1.1 ± 0.1	0.9 ± 0.1	0.7 ± 0.1	0.5 ± 0.1	0.3 ± 0.1	0.2 ± 0.1	–	–	–	–
1-Methyl-4-(6-methylhept-5-en-2-yl) cyclohexa-1,4-diene^®^-	32.28	0.58 ± 0.04	0.4 ± 0.1	0.3 ± 0.1	0.2 ± 0.1	0.1 ± 0.1	–	–	–	–	–	–
Pentadecane	32.59	0.22 ± 0.02	0.3 ± 0.1	0.4 ± 0.1	0.6 ± 0.1	0.8 ± 0.1	1.1 ± 0.1	1.4 ± 0.1	1.7 ± 0.1	2.0 ± 0.2	2.3 ± 0.2	2.6 ± 0.2
β-Bisabolene	32.80	0.18 ± 0.02	0.1 ± 0.1	–	–	–	–	–	–	–	–	–
α-Calacorene	34.08	0.19 ± 0.02	0.1 ± 0.1	–	–	–	–	–	–	–	–	–
Hexadecane	36.58	0.13 ± 0.02	–	–	–	–	–	–	–	–	–	–
Heptadecane	40.37	0.43 ± 0.03	0.36 ± 0.1	–	–	–	–	0.22 ± 0.1	0.16 ± 0.1	0.11 ± 0.1	–	–
Octadecane	43.98	0.35 ± 0.03	0.22 ± 0.1	0.15 ± 0.1	–	–	–	0.24 ± 0.1	0.18 ± 0.1	0.15 ± 0.1	–	–

^1^ For brevity, only five representative adulteration levels (10%, 20%, 30%, 40%, and 50%) are shown. The intermediate levels (5%, 15%, 25%, 35%, and 45%) were also analyzed and included in all chemometric models; however, the differences in volatile compound abundances between consecutive 5% increments were minimal (typically within measurement standard deviation) and are therefore not presented individually to avoid table overcrowding. Complete datasets are available upon reasonable request. ^2^ Invert sugar.

First, matrix effects play a significant role. The addition of sugar syrups alters the physicochemical properties of the sample, including viscosity, ionic strength, and water activity. These changes affect the partitioning of volatile compounds between the sample matrix and the headspace, thereby influencing extraction efficiency. This explains why some compounds (e.g., D-limonene, 1-nonanol, pentadecane) actually increase with adulteration—they may have higher affinity for the modified matrix or experience less competition for headspace partitioning.

Second, competitive adsorption on the SPME fiber may occur, as sugar components compete with volatile compounds for limited adsorption sites on the fiber coating. This effect would be compound-specific depending on each analyte’s affinity for the DVB/CAR/PDMS fiber.

Third, specific molecular interactions, particularly hydrogen bonding between sugar hydroxyl groups and oxygen-containing volatiles (alcohols, aldehydes), can retain certain compounds in the matrix, reducing their headspace concentration. This explains why linalool and octanal show steeper declines compared to non-polar hydrocarbons.

Importantly, rigorous quality control measures—including randomized run order, blank runs between samples, monitoring of QC samples, and triplicate analyses—confirm that these observations reflect genuine sample differences rather than analytical artifacts. The consistency of trends across multiple compounds and concentration levels further supports the reliability of the findings.

**Figure 1:**
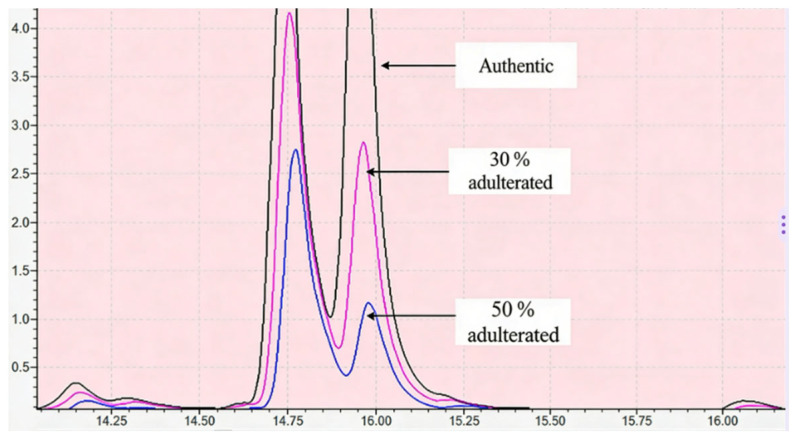
GC-MS Spectrum of linalool and nonanal in authentic honey, 30%, and 50% adulterated.

**Figure 2:**
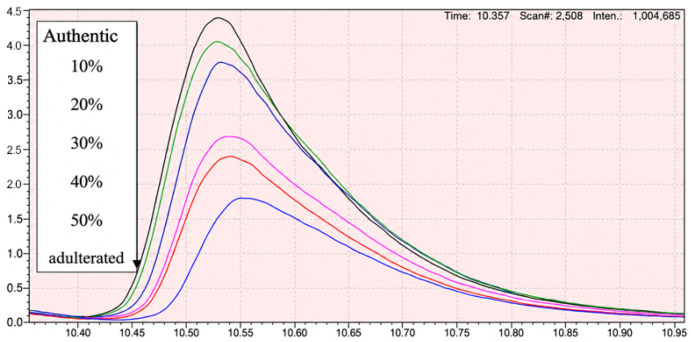
GC-MS Spectrum of octanal in authentic honey and different adulteration levels.

**Figure 3:**
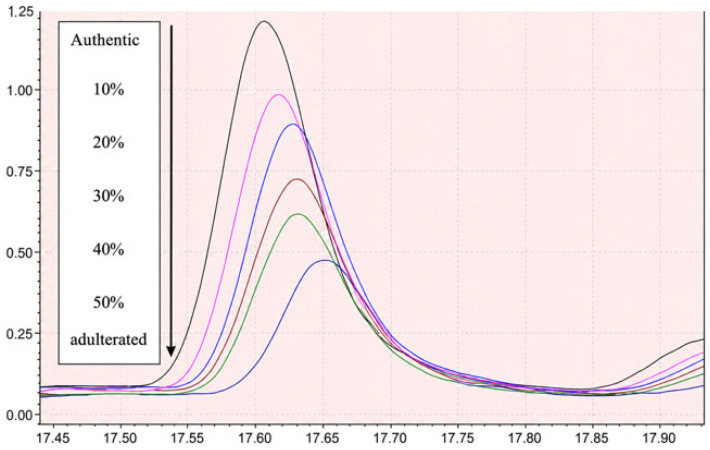
GC-MS Spectrum of α-terpineol in authentic honey and different adulteration levels.

Figure 1 depicts the chromatograms of linalool and nonanal. As shown, the levels of these two compounds, which are key markers of coriander honey, decreased with increasing adulteration levels. Additionally, Figure 2 and Figure 3 illustrate the concentrations of octanal and α-Terpineol, respectively, across different adulteration percentages, all demonstrating a reduction in compound levels as adulteration increases. According to the presented data, honey adulteration not only results in a decrease in volatile compounds in authentic honey but also reduces the total number of these compounds.

### 3.2. Chemometric Models and Classification

The minimum detectable adulteration level, defined as the lowest concentration at which 100% classification accuracy was achieved, was determined to be 5% (*w*/*w*) for both invert sugar and HFCS. At this level, all replicates were correctly classified by all four machine learning models (RF, SVM, KNN, and XGBoost). Table 3 presents the performance metrics of the SVM, RF, K-NN and XGBoost models for detecting honey adulteration. Among them, the XGBoost model showed the best results, achieving 95.83% accuracy, perfect precision (1.00) for authentic honey and 0.93 for adulterated honey, along with equally high recall values (1.00 for authentic and 0.9350 for adulterated honey). To ensure more reliable performance estimation given the dataset size (n = 69), all models were additionally assessed using 10-fold stratified cross-validation. As shown in Supplementary Figure S1, the results were consistent with the hold-out validation, with XGBoost achieving the highest mean accuracy of 95.4 ± 2.1%, followed by SVM (94.7 ± 2.8%). These findings confirm the robustness of the developed classification models.

XGBoost is a powerful ensemble learning technique that creates a single, highly accurate predictive model by combining the outputs of multiple weak learners. Known for its efficiency in managing missing data and large datasets, it leverages parallel processing to enhance computational speed and performance [20]. Although extensively utilized across various fields—including finance, healthcare, e-commerce, transportation, industry, and meteorology—its application in origin traceability remains relatively limited [25].

SVMs are advanced supervised classification algorithms that identify optimal hyperplanes for separating different classes. These hyperplanes are positioned to maximize the margin—the distance between the separating plane and the closest data points from each class, referred to as support vectors. Maximizing this margin enhances the model’s ability to accurately predict unseen data. SVMs are especially effective for nonlinear classification through the use of kernel functions, making them well-suited for complex spectral datasets. Due to their robustness and predictive accuracy, they have found broad applications in disciplines such as agriculture and medicine [26]. In this study RF could identify authentic honey samples from adulterated ones with an accuracy of 95%. In a 2024 study, Liu et al. employed data fusion combining Fourier transform mid-infrared (FT-MIR) and Raman spectroscopy to classify 142 honey samples from five distinct origins. By integrating a Particle Swarm Optimization (PSO) approach with an SVM model using a feature-level fusion method, they attained a test accuracy of 95.28%, demonstrating a dependable strategy for honey origin identification. However, this approach can be costly, demands skilled personnel, and is sensitive to sample variability and spectral interference, often requiring complex instrumentation and longer measurement times for consistent results [27].

RF, developed in the early 2000s, is a powerful yet simple machine learning approach that integrates the predictions of multiple decision trees to minimize overfitting and enhance accuracy [28]. In this study, the RF model attained a classification accuracy of 94.17% effectively differentiating among the honey samples, as detailed in Table 3. This result is consistent with previous research, such as the work by de Santana et al. (2019), where RF proved highly effective in detecting adulteration across various food products [28]. In that case, RF outperformed PLS-DA and performed on par with or better than SIMCA, with all samples included in the external validation without exclusions.

**Figure 4:**
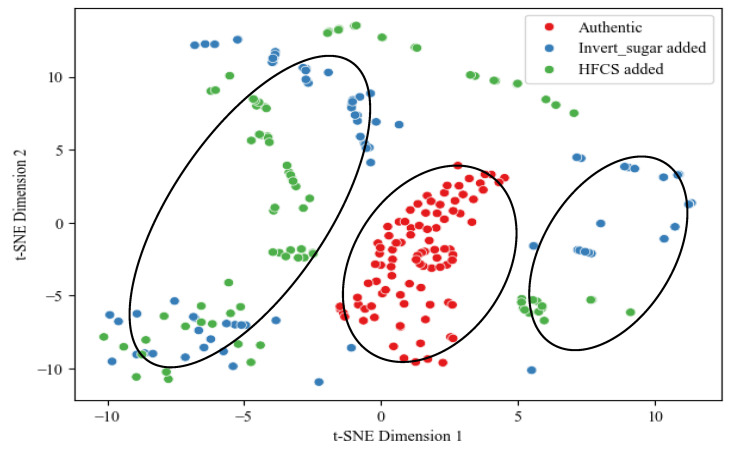
Dimensionality reduction results of GC-MS data by t-SNE.

KNN is a basic yet effective classification technique commonly applied when the underlying data distribution is unknown or difficult to define. The approach operates on the principle that samples close to one another in the feature space share similar class labels. In practice, KNN assigns a class to a new sample based on the majority class among its *k* nearest neighbors within the dataset [29]. In the current study, KNN achieved 94.17% accuracy in detecting authentic honey from adulterated samples. Also, sensitivity and specificity of this model were 91% and 95.5% respectively as shown in Table 3. In one study conducted in 2023 with Al-Awadhi and Deshmukh, honey adulteration detection was done using near-infrared combined with chemometrics including KNN. As their results showed, this model achieved accuracy of 77% in adulteration detection [30].

t-SNE is a nonlinear dimensionality reduction technique widely used for visualizing high-dimensional data in a low-dimensional space, typically two or three dimensions. Similar to the way methods like KNN rely on local neighborhood relationships, t-SNE focuses on preserving the local structure of the data—meaning points that are close in the original high-dimensional space remain close in the reduced space. The algorithm works by converting distances between points into probabilities that represent similarities, then arranging points in the low-dimensional map so that these probabilities are as consistent as possible with the original data. This makes t-SNE particularly effective for identifying and visually separating clusters or patterns within complex datasets, such as spectral profiles or classification results in food authentication studies [31]. As illustrated in Figure 4, the t-SNE method effectively achieved clear clustering and separation between the authentic honey samples and the two types of adulterated samples, invert sugar and HFCS. This result demonstrates the high capability of t-SNE in visualizing data structures and effectively distinguishing between different sample groups, thereby confirming the model’s strength and reliability in the classification process. However, t-SNE was unable to achieve clear separation between the two types of adulterated samples. This limitation is primarily attributed to the considerable similarity in their constituent sugars, as both invert sugar and HFCS are mainly composed of glucose and fructose in varying proportions. As a result, their chemical profiles overlap, making precise discrimination between these adulterants challenging for unsupervised clustering methods such as t-SNE. Although t-SNE is an unsupervised exploratory technique and not a formal validation method, it provided useful visualization of the clustering tendency between authentic and adulterated samples A study utilized FT-NIR spectroscopy to characterize leaves, petioles, and stems of three medicinal plants—Chamomile, Ginseng, and Quebra-pedras—commonly used in tea preparations. Using cluster analysis, the researchers assessed FT-NIR’s capability to distinguish among plant types, finding that t-SNE provided the highest discriminatory performance. Spectral deconvolution revealed 15 key vibration bands with strong characterization potential, highlighting FT-NIR as an effective technique for detecting plant-based adulteration in teas [32].

## 4. Discussion

**Figure 5:**
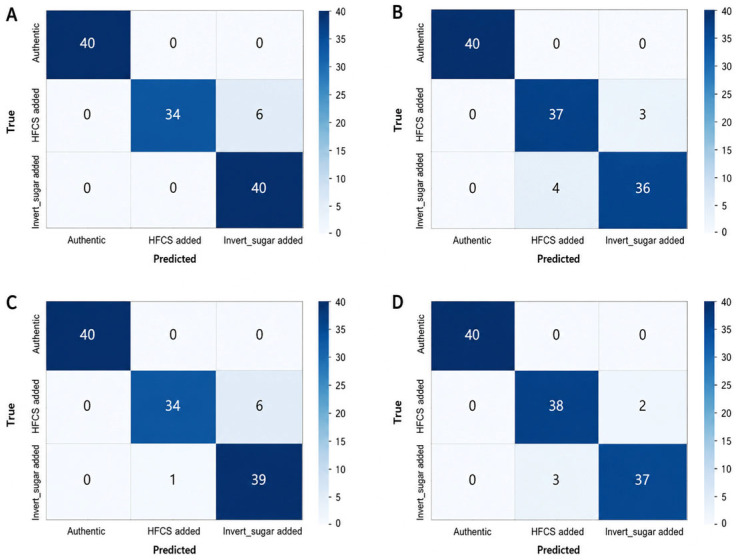
Confusion matrixes (**A**) SVM; (**B**) RF; (**C**) KNN; (**D**) XGBoost.

This study demonstrates the successful application of HS-SPME coupled with GC-MS and advanced chemometric methods for detecting adulteration in coriander honey. The results reveal a clear decline in both the concentration and diversity of volatile compounds as the percentage of adulterants, including invert sugar and HFCS, increases. This trend corroborates findings from other research on honey adulteration, highlighting the detrimental impact of sugar syrups on honey’s chemical profile and authenticity. (Table 4). Comparison of the four machine learning models revealed distinct advantages and limitations for honey adulteration detection. XGBoost demonstrated superior performance across all metrics, achieving the highest accuracy (95.83%) with excellent balance between precision and recall (F1-scores: 1.00, 0.94, and 0.94 for authentic, IS, and HFCS samples respectively). This confirms that gradient boosting algorithms are particularly effective at capturing complex, non-linear relationships in volatile profiling data, aligning with Wu and Wang [33] who reported XGBoost superiority for food authentication applications. SVM showed comparable overall accuracy (95.00%) with perfect precision (1.00) for HFCS samples, but lower recall (0.85) for this class, reflecting a trade-off between precision and recall when distinguishing HFCS from authentic honey; nevertheless, its performance was consistent with Lu et al. [34] who achieved 95.28% accuracy using SVM with data fusion approaches. Random Forest provided balanced performance across all classes (94.17% accuracy) with the important advantage of built-in feature importance identification, making it valuable for discovering key volatile markers, as previously noted by de Santana et al. [28] in food adulteration studies. KNN matched RF’s accuracy (94.17%) with excellent sensitivity for invert sugar (0.98), but showed lower sensitivity for HFCS (0.85) and greater sensitivity to feature scaling; despite these limitations, it significantly outperformed the 77% accuracy reported by Al-Awadhi and Deshmukh [30] for honey detection using similar approaches. These performance differences reflect each algorithm’s underlying mathematical approach—XGBoost’s gradient boosting captures non-linear interactions, SVM’s kernel trick handles high-dimensional spaces, RF’s ensemble averaging provides robustness, and KNN’s distance-based logic offers simplicity. Overall, while all four models proved effective for adulteration detection, the choice depends on the specific application: XGBoost is recommended when maximum accuracy is required, SVM excels with high-dimensional spectral data, RF provides superior interpretability for marker identification, and KNN offers a simple, rapid screening option with minimal computational requirements. This comprehensive comparison underscores the importance of model selection for honey authentication applications and provides a framework for researchers to choose the most appropriate algorithm based on their specific priorities. Furthermore, Figure 5 presents the confusion matrixes for the tested models, which provide clear evidence of the reliability and precision of the classifiers in distinguishing between authentic and adulterated honey samples (Figure 5A: SVM, Figure 5B: RF, Figure 5C: KNN and Figure 5D: XGBoost). As shown in Figure 5, the majority of misclassifications occurred between invert sugar and HFCS adulterated samples, which is expected given their similar sugar composition and consequent overlapping effects on the volatile profile. Meanwhile, dimensionality reduction by t-SNE illustrated excellent discrimination between authentic and adulterated samples, validating its utility for pattern recognition in spectral data. From a chemical perspective, the identified volatile compounds show distinct behaviors during adulteration. Terpenes: Linalool declines steeply due to hydrogen bonding with sugar hydroxyls, while non-polar D-limonene increases from reduced competition for SPME fiber sites. Aldehydes (octanal, nonanal) decrease linearly due to dilution and increased viscosity, serving as robust markers since they are absent in sugar syrups. Alcohols: 1-octanol decreases but 1-nonanol and 1-decanol increase, suggesting chain-length-dependent matrix affinity. Alkanes (pentadecane) consistently increase due to preferential headspace partitioning. Compounds disappearing at 5–20% adulteration (pyrazine, benzaldehyde, α-terpineol, and β-bisabolene) act as binary screening indicators. This confirms matrix effects—not dilution alone—govern volatile behavior, justifying linalool, octanal, nonanal, D-limonene, and pentadecane as authenticity markers. The proposed method successfully detected adulteration at concentrations as low as 5% (*w*/*w*) for both invert sugar and HFCS, demonstrating high sensitivity for authenticity assessment. This detection threshold is comparable or superior to other reported methods for honey adulteration detection using volatile profiling. Future studies should extend this approach to other honey types and include larger sample collections from diverse geographical origins to further validate method robustness.

## 5. Limitations

One of the primary limitations of the present study is the relatively small number of independent authentic coriander honey samples (n = 3). However, they still represent a narrow range of natural variability in terms of seasonal effects, beekeeping practices, floral nectar composition, and micro-geographical factors. Consequently, the adulterated samples were prepared from a representative authentic honey matrix. While this controlled experimental design allowed precise evaluation of the impact of invert sugar and HFCS adulteration levels on the volatile profile and facilitated high classification performance, the machine learning models were essentially trained to differentiate a limited authentic composition from its laboratory-prepared dilutions. This may lead to an overestimation of model generalizability when applied to entirely new authentic coriander honey samples from broader origins and conditions. Future studies should therefore prioritize the collection and analysis of a substantially larger set of authentic samples (at least 15–20 independent batches from multiple farms, seasons, and regions) to better validate the robustness and real-world applicability of the proposed HS-SPME-GC-MS and chemometric approach. Furthermore, because a semi-quantitative approach was adopted, formal recovery experiments and matrix effect validation were not conducted. This should be considered when interpreting the absolute changes in individual volatile compounds.

## 6. Conclusions

This study demonstrates that HS-SPME-GC-MS volatile profiling combined with chemometric analysis provides a rapid, sensitive, and reliable approach for detecting adulteration in Iranian coriander honey. The method successfully quantified changes in volatile compounds and differentiated authentic from adulterated samples with high classification accuracy. Regarding the modeling methodology, the comparative evaluation of four machine learning algorithms (SVM, RF, KNN, and XGBoost) revealed that ensemble methods, particularly XGBoost, are most effective for handling complex volatile profiling data, achieving 95.83% accuracy. The results demonstrate that supervised learning algorithms can successfully distinguish not only between authentic and adulterated samples but also, to a lesser extent, between different adulterant types despite their chemical similarity. Feature importance analysis identified linalool, octanal, and nonanal as key markers for coriander honey authenticity, providing interpretable insights into the chemical basis of discrimination. This comparative approach establishes a framework for algorithm selection in future food authentication studies, with XGBoost recommended for maximum accuracy, RF for marker identification, and KNN for rapid screening applications. However, the findings are currently specific to coriander honey from Iran and the generalizability of the proposed model is constrained by the limited number of authentic coriander honey sample (n = 3) used in this study; therefore, future research focusing on external validation on larger and independently collected authentic honey from diverse geographical origins is necessary before this approach can be confidently developed for routine authenticity testing.

## Figures and Tables

**Table 1:** Profile of volatile compounds in honey determined by GC-MS.

RT ^1^	Volatile Compounds	Kovats Index (KI) ^2^	
		Exp.	Lit.
4.30	Hexane, 2,4-dimethyl-	824	822
6.81	Nonane	900	900
7.18	Pyrazine, 2,5-dimethyl-	910	915
8.91	Benzaldehyde	957	961
10.01	β-Myrcene	987	991
10.40	Octanal	998	1000
11.51	D-Limonene	1024	1030
12.38	1,3,6-Octatriene,3,7-dimethyl-, (Z)-	1044	1041
12.85	2-Octenal, (E)	1054	1062
13.25	2-Octen-1-ol, (E)	1063	1069
13.47	1-Octanol	1068	1070
14.74	Linalool	1098	1098
14.96	Nonanal	1102	1102
18.02	1-Nonanol	1169	1172
18.67	3,6-Dimethyl-2,3,3a,4,5,7a-hexahydrobenzofuran	1183	1192
19.07	α-Terpineol	1192	1197
19.61	Decanal	1203	1203
21.28	(-)-Carvone	1240	1240
22.56	Nonanoic acid	1268	1278
22.65	1-Decanol	1270	1272
23.99	Tridecane	1299	1300
24.23	Undecanal	1304	1310
26.17	Naphthalene,1,2-dihydro-1,1,6-trimethyl-	1348	1355
28.39	Tetradecane	1398	1400
28.68	Tetradecanal	1405	1409
31.21	2H-Pyran-2-one,5,6-dihydro-6-pentyl-	1465	1477
31.67	Benzene, 1-(1,5-dimethyl-4-hexenyl)-4-methyl-	1476	1482
32.28	-1-Methyl-4-(6-methylhept-5-en-2-yl)cyclohexa-1,4-diene ^®^	1491	1480
32.59	Pentadecane	1498	1500
32.80	β-Bisabolene	1503	1506
34.08	α-Calacorene	1535	1542
36.58	Hexadecane	1598	1600
40.37	Heptadecane	1698	1700
43.98	Octadecane	1798	1800

^1^ Retention Time (min); ^2^ KI: KI (Exp.) = experimental Kovats index; (Lit.) = literature Kovats index (using NIST libraries).

**Table 3:** Adulteration detection model report.

Model	Evaluation Metric	Sample		
		Authentic	Inver Sugar Added	HFCS Added
RF	Precision	1	0.9231	0.9024
	Recall	1	0.9000	0.9250
	F1-score	1	0.9114	0.9136
	Sensitivity	1	0.9000	0.9250
	Specificity	1	0.9625	0.9500
	Accuracy = 94.17%			
XGBoost	Precision	1	0.9487	0.9268
	Recall	1	0.9250	0.9500
	F1-score	1	0.9367	0.9383
	Sensitivity	1	0.9250	0.9500
	Specificity	1	0.9750	0.9629
	Accuracy = 95.83%			
SVM	Precision	1	0.8696	1
	Recall	1	1	0.8596
	F1-score	1	0.9250	0.9302
	Sensitivity	1	1	0.8596
	Specificity	1	0.9250	1
	Accuracy = 95.00%			
K-NN	Precision	1	0.8667	0.9714
	Recall	1	0.9750	0.8500
	F1-score	1	0.9250	0.9176
	Sensitivity	1	0.9750	0.8500
	Specificity	1	0.9250	0.9875
	Accuracy = 94.17%			

**Table 4:** Summarizes studies on honey adulteration.

**Technique**	**Chemometrics Model**	**Accuracy/Performance**	**Reference Study**
FT-MIR	SVM/PLSR	98%	[35]
GC-MS	LDA	93.5%	[36]
Vis-NIRS	LDL	100%	[37]
ICP-MS	PNN/PLS	73–95%	[38]
Emission—Excitation Spectra	PLS-DA	70%	[21]
HS-SPME-GC-MS	XGBoost	95.83%	Our Study

## Data Availability

The original contributions presented in this study are included in the article/supplementary material. Further inquiries can be directed to the corresponding authors.

## Supplementary Materials

The following supporting information can be downloaded at https://www.mdpi.com/article/10.3390/foods15111988/s1, Figure S1: Performance of the four machine learning models evaluated using 10-fold stratified cross-validation (mean accuracy ± standard deviation). Table S1: Method Optimization and Validation; Table S2: Analytical performance characteristics of the developed HS-SPME-GC-MS method.

## Abbreviations

GC-MS	Gas Chromatography–Mass Spectrometry
HS-SPME	Headspace Solid-Phase Microextraction
VOC	Volatile Organic Compound
t-SNE	t-distributed Stochastic Neighbor Embedding
RF	Random Forest
XGBoost	eXtreme Gradient Boosting
SVM	Support Vector Machine
KNN	K-Nearest Neighbors
