# Supplementary material for: HS-SPME-GC-MS Coupled with Chemometrics for Detecting HFCS and Invert Sugar Adulteration in Coriander Honey
Source: Foods. 2026 Jun 3;15(11):1988. doi: 10.3390/foods15111988 (PMC13256990; doi:10.3390/foods15111988)
Supplement: Supplementary file 1 [file foods-15-01988-s001.zip › foods-4333949-supplementary.pdf]

Table S1: Method Optimization and Validation

Quality Control Measure	Implementation	Purpose
**Retention Index (RI) Verification**	Calculated using C8-C24 alkane standard mixture under identical conditions	Ensures correct compound identification independent of retention time drift
**System Suitability Test**	Injection of alkane standard mixture before each batch	Monitors chromatographic performance (peak shape, resolution)
**Blank Runs**	Empty vial analyzed between samples	Monitors carryover and fiber contamination
**Fiber Conditioning**	Pre-conditioning at 270°C for 60 min before each analysis day	Ensures consistent fiber performance
**Triplicate Analysis**	All samples analyzed in three replicates	Assesses analytical variability
**Relative Standard Deviation (RSD) Monitoring**	RSD calculated for all compounds across replicates	Compounds with RSD > 20% were flagged and reviewed

Table S2: Analytical performance characteristics of the developed HS-SPME-GC-MS method

Compound	LOD (μg/g)	LOQ (μg/g)	Linear Range (μg/g)	R^2
Linalool	0.02	0.07	0.1-50	0.997
Octanal	0.05	0.15	0.2-50	0.995
Nonanal	0.03	0.10	0.1-50	0.996
Decanal	0.08	0.25	0.5-50	0.994
D-Limonene	0.12	0.40	0.5-50	0.992

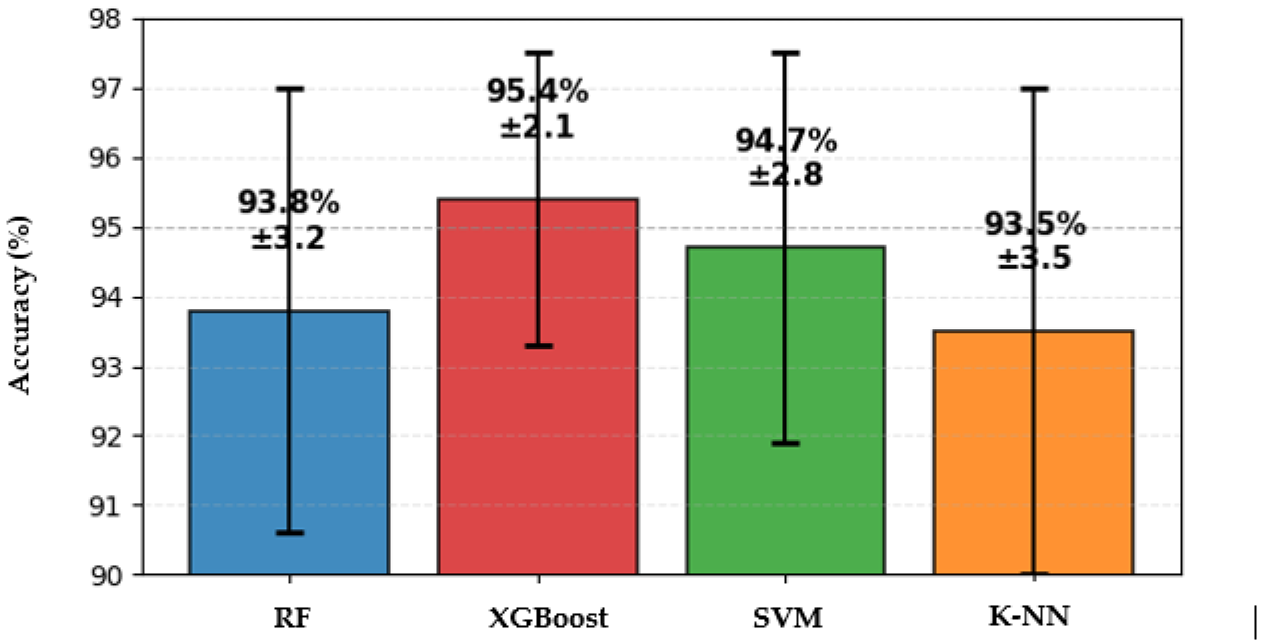

Figure S1. Performance of the four machine learning models evaluated using 10-fold stratified cross-validation (mean accuracy ± standard deviation).
